# Arthroscopic Suprapectoral Biceps Tenodesis for Isolated Biceps Tendinopathy: Results From 23 Patients

**DOI:** 10.7759/cureus.58912

**Published:** 2024-04-24

**Authors:** Apostolos Polyzos, Apostolos Gantsos, Vasileios Soranoglou, Vasileios A Kontogeorgakos, Alexandros Eleftheropoulos

**Affiliations:** 1 Orthopaedic Surgery, National and Kapodistrian University of Athens School of Medicine, Athens, GRC; 2 Orthopaedic Surgery, General Hospital of Naoussa, Naousa, GRC; 3 Orthopaedics and Traumatology, Athens General Hospital "G. Gennimatas", Athens, GRC; 4 Orthopedics, Attikon University General Hospital, Athens, GRC; 5 Orthopaedics and Traumatology, General Hospital of Naoussa, Naousa, GRC

**Keywords:** long head of the biceps tendon, shoulder arthroscopy, biceps tendinopathy, suprapectoral, biceps tenodesis

## Abstract

Introduction: Pathology affecting the long head of the biceps tendon (LHB) is a common cause of shoulder pain. When conservative treatment fails to resolve symptoms, surgical treatment is the modality of choice. The literature describes many arthroscopic and open techniques using different implants. However, no consensus exists on which procedure yields the greatest improvement. The purpose of this study was to evaluate the effectiveness and safety of arthroscopic suprapectoral biceps tenodesis for treating isolated LHB pathology.

Materials and methods: We present a case series of 23 patients with isolated LHB pathology who were treated with arthroscopic suprapectoral tenodesis between 2016 and 2022. All surgeries were performed by the same senior surgeon, and patients were assessed preoperatively and one year after the procedure, using the simple shoulder test (SST), Constant score (CS), and visual analog scale (VAS) by the senior surgeon. Statistical analysis was performed using the Wilcoxon Signed Rank test, with significance defined as p < 0.05.

Results: The CS improved from 68.52 (SD = 1.59) to 98 (SD = 7.1; p < 0.001), the SST improved from 8.78 (SD = 0.998) to 11.21 (SD = 0.42; p < 0.001), and the VAS improved from 8.26 (SD = 0.54) to 0 (SD = 0; p < 0.001) at one-year follow-up. No complications were reported postoperatively or during the follow-up period.

Conclusions: Arthroscopic suprapectoral biceps tenodesis significantly improved outcomes at one-year follow-up and can be considered an effective and safe choice when treating LHB pathology.

## Introduction

The role of the long head of the biceps tendon (LHB) is not thoroughly understood, and its contribution to shoulder joint kinematics and humeral stability thus remains ambiguous [1]. The function of the LHB has mostly been investigated in cadaveric studies, with most of these studies concluding that the LHB contributes to stabilizing the humeral head in all directions and preventing abnormal translations. Cadaveric testing has the limitation of not producing the proper dynamics of movement, but measurements obtained through in vivo studies have low accuracy [2].

Lesions of the LHB due to tendinopathy are prevalent in adults and have been widely recognized as an important factor in shoulder pain and decreased function [3,4]. Tendinopathy is a disorder of the tendon and is usually chronic in terms of pain and limited function [5,6]. Tendinopathy has a complicated pathology, and the basis of the pathophysiology is unclear [6]. Apart from sophisticated molecular mechanisms that have been suggested to be the genesis of tendinopathy, mechanical overload and inflammation have also been proposed to be contributors [6,7]. The pathology of tendinopathy-related changes can be observed in any section of the tendon, including the intraarticular and the extraarticular, which are the most commonly affected [8]. These sections of the tendon are exposed to frequent load and friction and are the most common sites for degeneration, which is often concomitant with shoulder pathology [8-11].

Treatment of LHB lesions begins with conservative modalities such as nonsteroidal anti-inflammatory drugs, rest, physiotherapy, and corticosteroid injections under ultrasound guidance [12]. When conservative treatment fails to improve symptoms, surgical treatment should be an option [12,13]. Surgical treatment of biceps lesions includes biceps tenotomy and tenodesis. Both techniques use multiple approaches, and especially for tenodesis, a variety of fixation types are described [3]. The theoretical advantage of tenodesis is that the length-tension relation of the biceps muscle results in better functional and cosmetic outcomes [3,13]. In the literature, many variations have been described for performing biceps tenodesis (open or arthroscopic, supra- or subpectoral, onlay, or inlay techniques) [14]. Currently, some studies indicate that different techniques have similar functional outcomes [15,16]; however, others suggest that tenodesis, and especially the suprapectoral technique, is complicated by residual pain, which may lead to revision surgery [15]. In contrast, an open subpectoral approach is related to musculocutaneous nerve injury and iatrogenic humeral fracture [14-16].

With regard to surgical treatment, no consensus exists on which procedure yields the most improved outcomes [3]. Furthermore, LHB pathology is often treated with concomitant shoulder joint pathology, which poses possible confounding factors when clinical outcome scores are obtained after surgery [17,18]. The lack of validated specific patient scores for biceps pathology adds further difficulty in assessing LHB pathology-related pain and function [1,3,17,18].

We hypothesized that arthroscopic suprapectoral biceps tenodesis could be an effective and safe choice for treating biceps pathology. The purpose of this study was to assess the effect of arthroscopic suprapectoral biceps tenodesis in clinical and functional outcomes when treating isolated biceps tendinopathy.

## Materials and methods

From June 2016 to January 2022, we conducted a prospective case series study. Patients undergoing operative treatment for biceps tendonitis were prospectively consecutively examined between June 2016 and January 2022 and included in a database. The study began with the approval of the local ethics committee. All patients provided written informed consent before undergoing the procedure. Inclusion criteria for the study were age greater than 18 years and less than 65 years, magnetic resonance imaging (MRI) findings and clinical assessment positive for biceps tendinopathy, and conservative treatment for a minimum of three months that was unsuccessful. Exclusion criteria were patients with rotator cuff tears, superior labrum anterior and posterior lesions diagnosed in MRI study, glenohumeral instability, previous surgery for biceps tendinopathy or other shoulder pathology, and total proximal biceps rupture.

**Figure 1:**
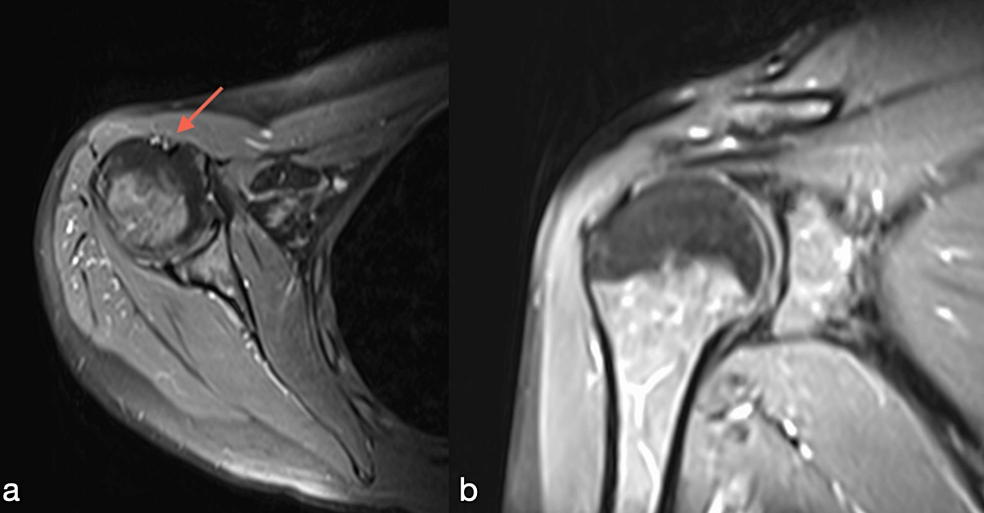
Magnetic resonance imaging of (a) the axial shoulder, showing biceps tendinopathy (arrow) and (b) the coronal shoulder showing no concominant rotator cuff pathology.

All patients were initially managed conservatively with physical therapy and pharmaceutical therapy using regular painkillers and nonsteroidal anti-inflammatory drugs. All patients underwent noncontrast MRI that led to a diagnosis of proximal biceps tendinopathy/tendinitis (Figure 1).

Every patient was examined preoperatively and one year postoperatively by the operating surgeon who was fellow trained in shoulder surgery. At the clinical examinations, the Constant score (CS) [19,20] and simple shoulder test (SST) results were obtained [19-22]. The CS was based on four sections: pain (0 to 15 points), activity level (0 to 20 points), range of motion (0 to 40 points), and strength (0 to 25 points, measured with the patient standing upright while using regular weights). The CS was adjusted for gender and age, and the sum of all sections was recorded for each patient [23]. The visual analog scale (VAS) was also used during the clinical visits [24].

Surgical technique

**Figure 2:**
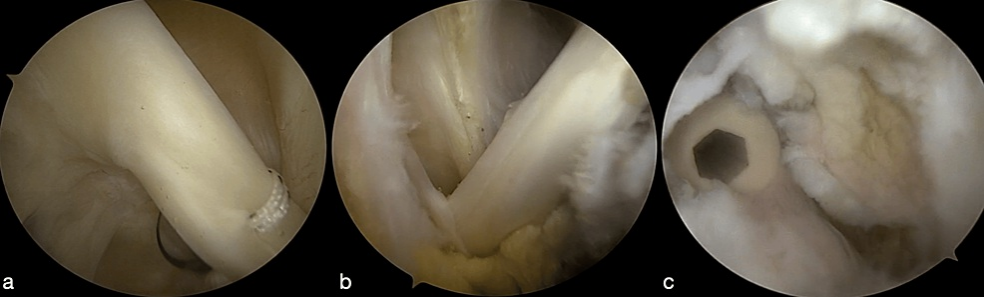
(a) Fiber wire passed laterally through the biceps tendon. (b) Biceps tendon secured away from groove before drilling. (c) Interference screw in the biceps tenodesis site. Fiber wire: Arthex Inc., Arthrex, USA

All operations were performed arthroscopically using the beach chair position. A standard posterior portal for shoulder arthroscopy was used to enter the joint. Diagnostic arthroscopy was performed in all cases, and it confirmed that there was no concomitant pathology. A standard anterior (lateral to the coracoid and below the coraco-acromial ligament) portal was established, followed by dynamic assessment of biceps tendon and regular assessment of all other anatomical structures. Furthermore, a penetrator was used to pass a loop 2-0 Tiger-wire through the biceps tendon as close to lateral as possible. Biceps tenotomy was performed after securing the tendon with the loop suture. The proximal stump of the tendon was contoured with regular shavers. In addition, a standard lateral portal was established (2-3 cm below the lateral edge of the acromion). Acromioplasty was performed using the lateral portal. With the arm placed in 20° of forward flexion, the bursae at the anterior aspect of the glenohumeral joint were prepared. The muscle fibers of the deltoid and fibers of the tendon of the pectoralis major were recognized. The biceps tendon was recognized just medial to the insertion of the pectorals major tendon, and an anterior-inferior accessory portal was established. The biceps tendon was secured away from the groove, and then an 8.5-mm noncannulated reamer was used to prepare the tenodesis site at the suprapectoral area for male patients; a 7.5-mm reamer was used for female patients. An 8-mm Swivelock tenodesis screw (Arthex Inc., Arthrex USA) was advanced to facilitate the tenodesis for male patients, while a 7-mm screw was used for female patients. The position of the screw was checked, and the remaining tendon above the tenodesis site was resected (Figure 2) [25].

The patients were discharged the following day, and a regular arm sling was used for three weeks. Passive elbow and shoulder motion was encouraged postoperatively from the first week until the fourth week. After the fourth week, patients were encouraged to actively use their elbow and shoulder. Resistance exercises and return to everyday activities were allowed three months postoperatively.

## Results

After the implementation of the inclusion/exclusion criteria, three patients were excluded from the study due to concomitant rotator cuff tears that needed surgical repair.

The final data set consisted of 23 patients and 23 shoulders eligible for the study. The mean age of the patients was 45 years old (SD = 7.7; range, 33-59) at the time of the intervention. There were seven (30.4%) male and 16 (69.6%) female patients. The mean body mass index of the patients was 23.7 (SD = 3.2; range, 18.9-30.5) before the procedure. The dominant arm was operated on in 18 (78.3%) of the 23 patients. Other than one female patient who had hypothyroidism (under treatment), none of the patients had comorbidities. The mean duration of symptoms for the patients before the operative treatment was eight months (SD = 3.5; range, 3-17). None of the patients reported any trauma before the operation (Table 1).

**Table 1:** Patients’ demographic data (N = 23). SD: Standard deviation

Characteristic	Value or quantity
Age, years (SD)	45 (7.7)
Sex	
Male	7
Female	16
Body mass index (SD)	23.7 (3.2)
Dominant arm operated (no. of patients)	18
Duration of symptoms, months (SD)	8 (3.5)
Comorbidities	1 case of hypothyroidism

For data analysis, we first used the Shapiro-Wilk test to assess CS values for normality. Shapiro-Wilk testing was used due to the small sample size, as determining the variable distribution was crucial for choosing the appropriate statistical method [26]. The results showed that the distribution of CS (both preoperatively and postoperatively) departed significantly from normality (preoperative: W = 0.86, p = 0.04; postoperative: W = 0.81, p < 0.001). Based on this outcome, a Wilcoxon signed-rank test [27] was used, and the results indicated that the postoperative CS at one year after the procedure (mean = 98, SD = 7.1) was significantly higher than the preoperative CS (mean = 68.52, SD = 1.59). The mean difference in scores was 29.48 (z = 276, p < 0.001).

The Shapiro-Wilk test was also used to test for normality of SST values. The test showed that the SST distribution (both preoperatively and postoperatively) departed significantly from normality (preoperative: W = 0.62, p < 0.001; postoperative: W = 0.51, p < 0.001). As with the previous variable, the Wilcoxon signed-rank test was used [27], and the results indicated that the postoperative SST at one year after the procedure (mean = 11.21, SD = 0.42) was significantly higher than the preoperative SST (mean = 8.78, SD = 0.998). The mean difference was 2.43 (z = 276, p < 0.001).

VAS values were tested for normality using the Shapiro-Wilk test. The test showed that the distribution of preoperative values departed significantly for normality (W = 0.54, p < 0.001), and the postoperative values were all 0. The Wilcoxon signed-rank test [27] indicated that postoperative VAS (mean = 0, SD = 0) was significantly lower than the preoperative VAS (mean = 8.26, SD = 0.54). The mean difference was 8.26 (z = 276, p < 0.001) (Table 2).

**Table 2:** Constant score, simple shoulder test, and visual analog scale preoperatively and in the one-year follow-up. SD: Standard deviation

Assessment	Preoperative	Postoperative (one-year follow-up)
Constant score (SD)	68.52 (1.59)	98 (7.1)
Simple shoulder test (SD)	8.78(0.998)	11.21 (0.42)
Visual analog scale, cm (SD)	8.26 (0.54)	0 (0)

All data were analyzed using IBM SPSS Statistics for Windows, Version 28 (Released 2021; IBM Corp., Armonk, New York, United States). No revision surgery was needed in any patient, and none of the procedures resulted in neural or vascular damage. In addition, none of the patients reported any complications concerning the procedure, and none of them reported any long-term or cosmetic complications (Popeye sign or other).

## Discussion

Our study showed that the CS, SST, and VAS after biceps tenodesis were significantly improved (all p < 0.001) after one year of follow-up. The CS is a tool for assessing shoulder function [28], and the SST is a valid, reliable, and sensitive test for assessing shoulder pain [21]. In addition, the VAS is a valid tool for evaluating chronic pain [24,29]. Patients in our study had better shoulder function and significantly lower pain levels one year after the procedure. The operated patients had no complications, no residual deformities such as Popeye sign, no neural or vascular damage, no surgical site infections, and no need for revision surgery [30].

This study suggests that arthroscopic suprapectoral biceps tenodesis using this particular technique is a safe treatment modality for biceps tendonitis. In addition, all patients were clinically improved one year after the procedure, which was reflected in the results for the functional and pain scores. The clinical improvement of patients was supported by the difference in the means of the scores tested being greater than the minimally clinically important difference (MCID) of shoulder outcomes [29]. In the literature, the MCID for the CS is reported to be between 8 and 10.4 [31,32] for different shoulder pathologies, and the SST MCID is reported to be 1.5 points [33]. Furthermore, the VAS MCID after biceps tenodesis has been reported to be 1.29 [29,34]. In our study, the mean differences of the CS, SST, and VAS were greater than the MCIDs reported in the literature.

The literature describes arthroscopic suprapectoral biceps tenodesis using different implant types. Several cadaveric studies have included comparisons of different tenodesis techniques and implants, showing similar properties and clinical outcomes [35]. In their systematic review and meta-regression, Aida et al. found that anchor constructs had inferior ultimate failure load, but if they were augmented with sutures, the total strength could be increased [35]. A cadaveric study by Vestermark et al. suggested that there were no significant differences between anchors with lasso loop and interference screw tenodesis [36]. A systematic review and meta-analysis of biceps tenodesis by Dekker et al. also found no significant biomechanical differences between fixation techniques [37]. In addition, in a recent systematic review and meta-analysis of different techniques used in biceps tenodesis, Jackson et al. showed no statistically significant difference between onlay and inlay techniques [14]. Both had improved clinical outcomes. In onlay techniques, the tendon is secured on the cortical surface, while inlay techniques are performed with an intraosseous tenodesis [14].

Cabarcas et al. found that arthroscopic suprapectoral biceps tenodesis with an anchor for biceps pathology had a statistically significant improvement in the CS (preoperative = 40 ± 11.6; six-month follow-up = 64.6 ± 11.9), which exceeded the reported MCID (6.9) of the study [10]. The VAS was also statistically significantly improved at the six-month follow-up (preoperative = 6.1 ± 2.4; 6-month follow-up = 3.1 ± 2.3), which was greater than the MCID (1.5) reported in the study [10]. Our study showed similar results at the one-year follow-up concerning CS and VAS improvement using suprapectoral tenodesis with a tenodesis screw.

In a study by Vitali et al., arthroscopic suprapectoral biceps tenodesis was used for the treatment of isolated biceps tendon pathology [38]. At one year of follow-up, the authors reported significant improvement in the CS and VAS (p < 0.05) in 60 patients. The study also reported no complications during the follow-up. These findings correlate with our study’s findings, as our results showed the same outcomes for the CS and VAS while having no complications using suprapectoral tenodesis.

In their network meta-analysis, Anil et al. included cohort studies that managed LHB pathology using open, arthroscopic, subpectoral, and suprapectoral techniques for tenodesis [3]. Their study concluded that tenodesis (open or arthroscopic) has better functional outcomes than tenotomy. In addition, they stated that suprapectoral tenodesis had statistically significant improved outcomes concerning the CS (p = 0.031), which correlates with our study's results.

Moreover, in our study, none of the patients reported any cosmetic complications in the tenodesis or biceps area, and none of the patients had any report indicating Popeye sign. This finding accords with previous reports [39,40] and especially in a meta-analysis of Level I randomized control trials by Belk et al. [39]. The latter authors compared biceps tenotomy with tenodesis and found that it reportedly had a higher rate of cosmetic deformity [39]. Additionally, in a study by Galdi et al., patients were given a specific biceps questionnaire before planned surgery [41]. Patients preferred tenodesis over tenotomy after using the questionnaire and the logistic regression of the study showed that female sex, amount of concern about cosmetic deformity, and significance of pain relief were predicting factors when patients chose the modality of treatment [41].

In our study, we made an effort to examine the results of the biceps tenodesis technique when treating biceps pathology in the absence of concomitant lesions. Thus, a potential strength of our study is that it only included patients with isolated biceps pathology. Biceps pathology is a common concomitant lesion in other shoulder pathologies, such as rotator cuff tears [42]. Assessment of clinical outcomes may have some confounding factors when other shoulder pathologies are being treated in the same procedure [11,42].

This study has several limitations. The absence of a control or comparison group with other techniques limits the level of evidence of data this study provides. Furthermore, this study included a small number of patients, as the primary surgeon carefully examined the patients to ensure they fully met the inclusion criteria and did not have any concomitant shoulder pathology. We performed acromioplasty during the tenodesis as an adherent part of our technique. This was due to the working space required for performing the technique at the anterior aspect of the shoulder. Although none of the patients had any impingement-related symptoms, our clinical outcomes include the possible benefits of an acromioplasty having been performed.

## Conclusions

In conclusion, arthroscopic suprapectoral biceps tenodesis with an interference screw is an effective modality of treatment for biceps tendinopathy. It provides significantly improved outcomes concerning functionality and pain as documented in the scores at the one-year follow-up. Furthermore, no patient reported any postoperative complications. Thus, arthroscopic suprapectoral biceps tenodesis appears to be a procedure that can relieve pain, improve functionality, and prevent notable complications in biceps surgery such as Popeye sign or other cosmetic complications at the biceps site.
